# The circadian rhythm of bladder clock genes in the spontaneously hypersensitive rat

**DOI:** 10.1371/journal.pone.0220381

**Published:** 2019-07-25

**Authors:** Yusuke Kimura, Masashi Honda, Ryo Sasaki, Tetsuya Yumioka, Hideto Iwamoto, Panagiota Tsounapi, Shuichi Morizane, Katsuya Hikita, Mitsuhiko Osaki, Futoshi Okada, Atsushi Takenaka

**Affiliations:** 1 Division of Urology, Tottori University Faculty of Medicine, Yonago, Japan; 2 Division of Pathological Biochemistry, Tottori University Faculty of Medicine, Yonago, Japan; 3 Chromosome Engineering Research Center, Tottori University Faculty of Medicine, Yonago, Japan; Karlsruhe Institute of Technology, GERMANY

## Abstract

Circadian expression rhythms of clock gene products in the bladder are reportedly hindered by clock gene abnormalities. However, the role of clock gene products in various pathological lower urinary tract conditions is unknown. The present study examined the relationship between clock genes and voiding dysfunction in spontaneous hypertensive rats (SHR). The voluntary voiding behavior study using metabolic cages was performed in 18-weeks old male Wistar rats (control group, n = 36) and SHR (SHR group, n = 36) under 12-h light/12-h dark conditions. Bladders were harvested every 4 h at six time points (n = 6 for each time point for each group), and we analyzed the messenger RNA (mRNA) expression of several clock genes: period 2 (*Per2*), cryptochrome 2 (*Cry2*), brain and muscle aryl hydrocarbon receptor nuclear translocator-like protein 1 (*Bmal1*), circadian locomotor output cycles kaput (*Clock*), nuclear receptor subfamily 1, group D, member 1 (*Rev-erbα*), mechanosensors: transient receptor potential vanilloid channel 1 (*TRPV1*), *TRPV4*, *Piezo1*, and vesicular nucleotide transporter (*VNUT*) using real-time polymerase chain reaction. Though 24-h urination frequency for both light and dark periods was significantly higher in the SHR group, urine volume per voiding was significantly lower versus control. In controls, urine volume per voiding was significantly lower during the dark period (active phase) than the light period (rest phase); this parameter did not significantly differ between active and rest phases for SHR. SHR bladders showed significantly higher expression of *Cry2* and *Clock* during the active phase compared to controls. In the SHR group, *TRPV1*, *TRPV4*, *Piezo1*, and *VNUT* mRNA levels were significantly higher during the active phase compared to the control group. We speculate that Cry2 and Clock may be contributing factors in the decrease of bladder capacity during the active phase in SHR through increase of *TRPV1*, *TRPV4*, *Piezo1*, and *VNUT* expression, but further research will be necessary to elucidate the precise mechanisms.

## Introduction

Circadian rhythms are under the control of clock gene products, which are present in most cells and organs of mammals. The suprachiasmatic nucleus of the brain works as a master pacemaker [1] and synchronizes peripheral clock gene rhythms that exist in multiple tissues of the whole body, such as the lungs, liver, kidneys, and bladder [2, 3]. The mechanisms of these synchronizing pathways are likely to involve both hormonal and neuronal mechanisms; however, other pathways are not completely understood. The first gene identified to be involved in suprachiasmatic nucleus pacemaking was circadian locomotor output cycles kaput (*Clock*) [4], followed by period (*Per*) *1–3*, cryptochrome (*Cry*) *1–2*, brain and muscle aryl hydrocarbon receptor nuclear translocator-like protein 1 (*Bmal1*), neural per-arnt-sim (PAS) domain protein-2, and nuclear receptor subfamily 1, group D, member 1 (*Rev-erbα*). Clock gene products constitute a circadian mechanism based on a self-sustaining transcription-translation feedback loop [5]. Rotation of this feedback loop takes about 24 h and forms rhythms in various biological processes in the body. In the bladder, several groups have reported that lower urinary tract functions are regulated by clock genes [6, 7].

Overactive bladder (OAB) is defined as urinary urgency with or without urge incontinence, usually co-existing with frequency and nocturia. Hypertension may affect pelvic arterial blood flow, resulting in the loss of smooth muscle function in the bladder and consequent loss of bladder compliance [8]. Such changes may result in lower urinary tract symptoms, including OAB. In particular, spontaneously hypertensive rats (SHR) have been reported to be a suitable model for various diseases, including metabolic syndrome, hypertension, and even OAB.

Transient receptor potential vanilloid (TRPV) channels are multifunctional sensors at the cellular level. They bind specific ligands and can be activated by chemical (osmolality and pH) or physical (mechanical stress, cold, heat, and voltage) stimuli [9]. TRPV1 is expressed in the epithelial cells lining the urothelium, interstitial cells, and in sensory nerve terminals. TRPV1 is reportedly required for detection of bladder stretch, which involves the stretch-evoked release of adenosine triphosphate (ATP) and nitric oxide [10]. In OAB, some studies have shown a significantly increased expression of *TRPV1* in tissue biopsies of bladder urothelium [11]. In the esophagus, expression of *TRPV1* has a diurnal rhythmic pattern, together with nerve growth factor and clock genes, such as *Bmal1*, *Per1*, *Per2*, and *Cry2* [12]. Moreover, TRPV4 senses distension of the bladder urothelium, which is converted into an ATP signal in the micturition reflex pathway during urine storage. Thus, TRPV4 may contribute to bladder function, especially the mediation of bladder distention signals to primary afferent nerves during urine storage [13]. Vesicular nucleotide transporter (VNUT) is a novel member of an anion transporter family and is abundantly expressed in the bladder urothelium, which releases ATP by exocytosis when weakly stimulated. Nakagomi *et al*. have previously reported that urothelial VNUT-dependent ATP exocytosis is involved in urine storage mechanisms that promote relaxation of the bladder during the early stages of filling [14]. Piezo1 is present in the bladder urothelium and has a functional role of sensing extension of the bladder urothelium by means of a stretch-evoked Ca^2+^ influx and ATP release in mouse urothelial cells [15].

The circadian rhythm of clock gene expression in SHR has been reported in several organs. The abnormal circadian system reported in the liver of SHR [16] has been correlated with the fact that SHR are predisposed to metabolic disease. Other reports have suggested that temporal control of the physiology in SHR may be affected due to abnormal day/night rhythms in blood pressure [17] and aberrant sleeping patterns [18]. However, it is unknown whether clock gene products play a role in various pathological conditions in the lower urinary tract, such as OAB. Therefore, the aim of the present study was to examine the relationship between clock gene expression and bladder dysfunction in SHR.

## Materials and methods

### Animals

Six-week-old male Wistar rats (control; Japan SLC Inc., Hamamatsu, Japan) and SHR (Japan SLC Inc., Hamamatsu, Japan) were maintained in the Institute for Animal Experimentation of Tottori University under specific pathogen-free conditions with a 12-h light/12-h dark cycle (lights on from 07:00 to 19:00) at 23 ± 3°C and 50% ± 10% humidity and had free access to food (CE-2; CLEA Japan, Inc., Tokyo, Japan) and water. The time at which the light period began was set to zeitgeber time (ZT) 0, with the dark period beginning at ZT 12. This study was approved by the Tottori University Institutional Animal Care and Use Committee (Permission number: h29-Y-002) and complied with The American Physiological Society’s “Guiding Principles in the Care and Use of Animals”.

### Metabolic cage experiments

Upon reaching 18 weeks of age, conscious rats were individually placed in metabolic cages (Metabolica type MC-ST; Sugiyama-Gen, Tokyo, Japan) (n = 36 for each group) with free access to food and water to begin evaluation of voluntary voiding behavior. Voided urine from each rat was collected in a cup and placed on an electronic balance connected to a multiport controller (PowerLab; AD-Instruments, Pty Ltd., Castle Hill, Australia) and computer (R501C; ASUSTeK Computer Inc., Taipei, Taiwan) to record voiding frequency and volume. The results were analyzed using LabChart 8 software (PowerLab/8sp; AD-Instruments, Pty Ltd., Castle Hill, Australia) and then urine weight was converted to volume using the assumption that 1 g of urine corresponds to 1 mL. The parameters evaluated were the voided volume, voiding frequency, and urine volume per voiding (defines bladder capacity) over 24 h during both light and dark periods. Since rats are nocturnal [19], the dark period was considered as the active phase and the light period as the rest phase.

### RNA extraction and reverse transcriptase polymerase chain reaction (RT-PCR)

When metabolic cage experiments were completed, rats were anesthetized with pentobarbital (60 mg/Kg, intraperitoneal), and bladders were collected every 4 h at six time points (ZT 3, 7, 11, 15, 19, and 23) from both groups (n = 6 for each time point, total n = 36 for each group). Rats were euthanized by isoflurane/CO_2_ euthanasia without fasting. RNA in the bladder was measured by polymerase chain reaction (PCR) as previously reported [20]. In brief, bladder samples were homogenized and total RNA was extracted using an RNeasy Mini Kit (Qiagen, Hilden, Germany). The total RNA concentration was determined by measuring absorbance at 260 nm, and RNA quality was verified by 1% agarose gel electrophoresis with ethidium bromide. Total RNA was then reverse transcribed in a final volume of 11.5 μL containing 4 μL of 5 × standard buffer, 2 μL of 0.1 M dithiothreitol (dTT), 1 μL of Super Script II RNase H-Reverse Transcriptase (Invitrogen, Carlsbad, CA, USA), 2 μL of 10 mM Deoxynucleotide (dNTP) Mix (Promega, Madison, WI, USA), 1 μL of 50 pmol/μL Random Primer (Promega, Madison, WI, USA), 0.5 μL of 100 pmol/μL Oligo (dt) 15 Primer (Promega, Madison, WI, USA), and 1 μL of 40 U/μL ribonuclease inhibitor (Wako Pure Chemical Industries, Ltd., Osaka, Japan). Samples were then incubated at 37°C for 60 min, 95°C for 5 min, and cooled to 4°C for 5 min.

### Quantitative real-time PCR

Quantitative real-time PCR was performed in a final volume of 10 μL containing 4.1 μL of PCR grade water, 1 μL of Universal Probe Library probe (Roche, Tokyo, Japan), 0.2 μL each of forward and reverse primers (10 μM each), 2 μL of Light Cycler TaqMan Master (Roche, Tokyo, Japan), and 2.5 μL of complementary DNA (cDNA). The mRNA levels of *TRPV1* (GenBank, NM_031982.1), *TRPV4* (GenBank, XM_006249466.2), *VNUT* (GenBank, NM_001108613.1), *Piezo1* (GenBank, NM_001077200.2), *Rev-erbα* (GenBank, NM_001113422.1), *Per2* (GenBank, NM_031678.1), *Bmal1* (GenBank, NM_024362.2), *Cry2* (GenBank, NM_133405.2), and *Clock* (GenBank, NM_001289832.1) were assessed using a Light Cycler Fast Start DNA Master SYBR Green 1 RT-PCR assay (Roche, Tokyo, Japan) with ß-actin (GenBank, NM_031144.3) as the housekeeping gene (Table 1). Thermal cycle conditions were held at 95°C for 10 min followed by 45 cycles of 95°C for 30 s and 60°C for 1 min.

**Table 1:** Primers used for real-time PCR.

Gene	Accession no.	Primers
*TRPV1*	NM_031982.1	F: 5'-GCTCTGCTCCTGGACGTT-3'
		R: 5'-GGCAATGTGCAGTGCTGT-3'
*TRPV4*	XM_006249466.2	F: 5'-CCACCCCAGTGACAACAAG-3'
		R: 5'-GGAGCTTTGGGGCTCTGT-3'
*VNUT*	NM_001108613.1	F: 5'-CTTGCTCTGGGTGTACTACGTG-3'
		R: 5'-AGGGCCAGGACAAGGTCT-3'
*Piezo1*	NM_001077200.2	F: 5'-GACGCCTCACAAGGAAAGC-3'
		R: 5'-GGGCAGCATCTATGTCATCC-3'
*Rev-erbα*	NM_001113422.1	F: 5'-CGACCCTAGACTCCAACAACA-3'
		R: 5'-TGCCATTGGAGCTGTCACTA-3'
*Per2*	NM_031678.1	F: 5'-CAAAAGGAAGCCTCTGTTGC-3'
		R: 5'-GGTGGTGACAGACCTCACCT-3'
*Bmal1*	NM_024362.2	F: 5'-CTCCCCCTGATGCTTCCT-3'
		R: 5'-TGTCTGGAGTCCCTCCATTT-3'
*Cry2*	NM_133405.2	F: 5'-GGGAGCATCAGCAACACAG-3'
		R: 5'-GCTTCCAGCTTGCGTTTG-3'
*Clock*	NM_001289832.1	F: 5'-CGAGACAGCTGCTGACAAAA-3'
		R: 5'-TGAGACTCACTGTGTTTATACGATTG-3'
*βactin*	NM_031144.3	F: 5'-CCCGCGAGTACAACCTTCT-3'
		R: 5'-CGTCATCCATGGCGAACT-3'

*TRPV1*: transient receptor potential vanilloid channel 1; *TRPV4*: Transient receptor potential vanillodi channel 4; *VNUT*: vesicular nucleotide transporter; *Rev-erbα*: nuclear receptor subfamily 1, group D, member 1; *Per2*: period 2; *Bmal1*: brain and muscle aryl hydrocarbon receptor nuclear translocator-like protein 1; *Cry2*: cryptochrome 2; *Clock*: circadian locomotor output cycles kaput.

### Statistical analysis

All statistical analyses were performed using IBM SPSS Statistics for Windows version 23.0 (IBM Corp., Armonk, NY, USA). Student’s unpaired *t*-test was used for body and bladder weight data analysis. One-way analysis of variance (ANOVA) followed by Dunnett’s multiple comparison test was used for metabolic cage and PCR data analysis. A p<0.05 was considered statistically significant.

## Results

### Physiological parameters

**Fig 1:**
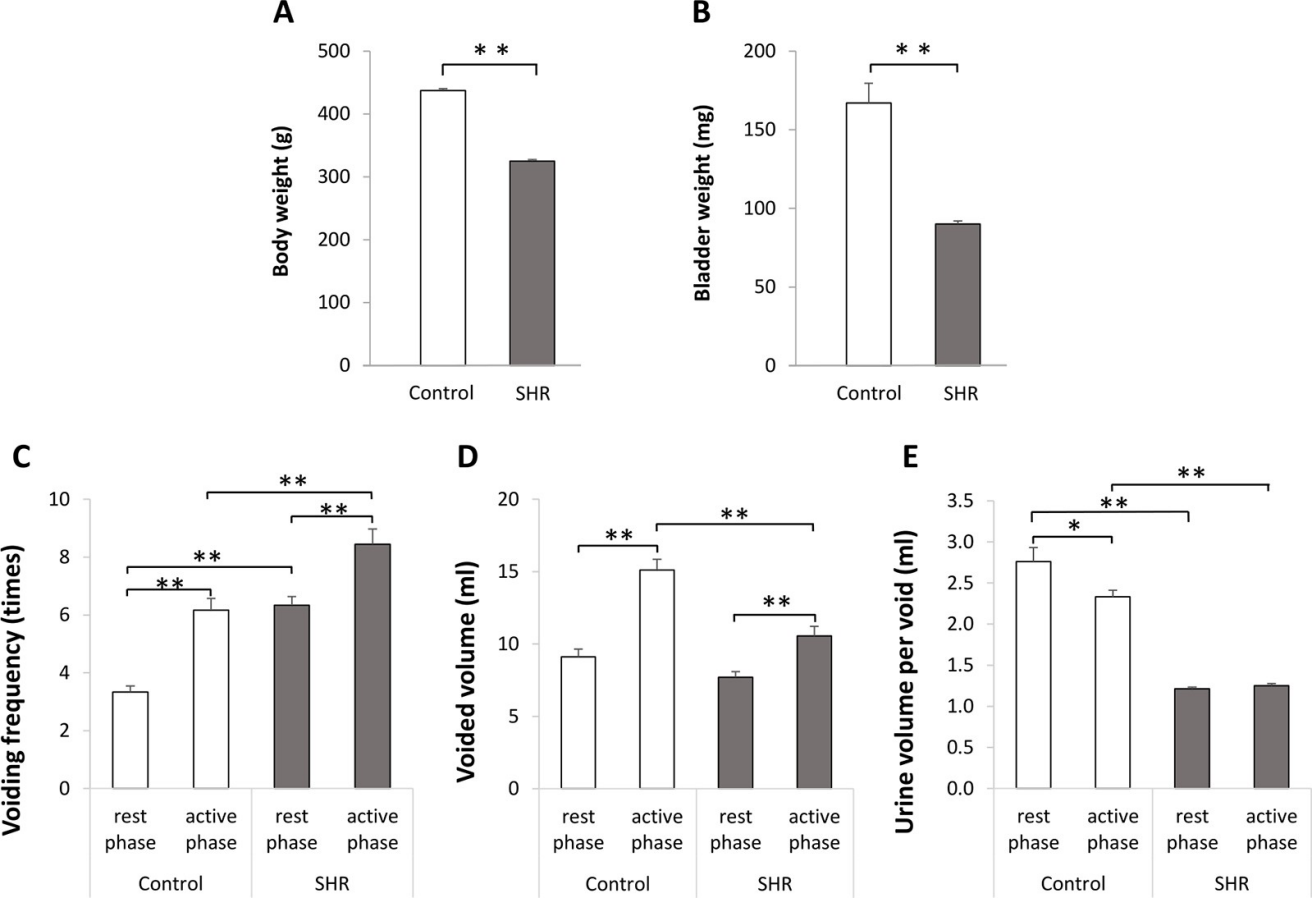
Body and bladder weight and results of voiding behaviors. **A:** Body weight in 18-week-old Wistar rats (control) and spontaneously hypertensive rats (SHR). **B:** Bladder weight in 18-week-old control and SHR. **C:** Voiding frequency in the rest and active phase in 18-week-old control and SHR under 12-h light/12-h dark cycle. **D:** Voided volume in the rest and active phase in 18-week-old control and SHR under 12-h light/12-h dark cycle. **E:** Urine volume per void in in the rest and active phase in 18-week-old control and SHR under 12-h light/12-h dark cycle. n = 36 in the control group; n = 36 in the SHR group. Student’s unpaired *t*-test was used for body and bladder weight data analysis. One-way analysis of variance (ANOVA) followed by Dunnett’s multiple comparison test was used for metabolic cage data analysis. *p<0.05, ** p<0.01.

Body and bladder weights were significantly lower in the SHR group than in the control (Fig 1A and 1B), while 24-h voiding frequency in both the rest and active phases was significantly higher in the SHR group (Fig 1C). Within both groups, the voiding frequency was significantly higher in the active than in the rest phase. Although the voided volume in the active phase was significantly higher than that in the rest phase for both groups, the 24-h urine volume in the active phase was significantly lower in the SHR group compared to the control group (Fig 1D). Urine volume per voiding over 24 h was significantly lower in the SHR versus control group in both the rest and active phases (Fig 1E). Moreover, while urine volume per voiding was significantly lower in the active than the rest phase for the control, this parameter was not significantly different between phases in the SHR.

### Clock gene expression

**Fig 2:**
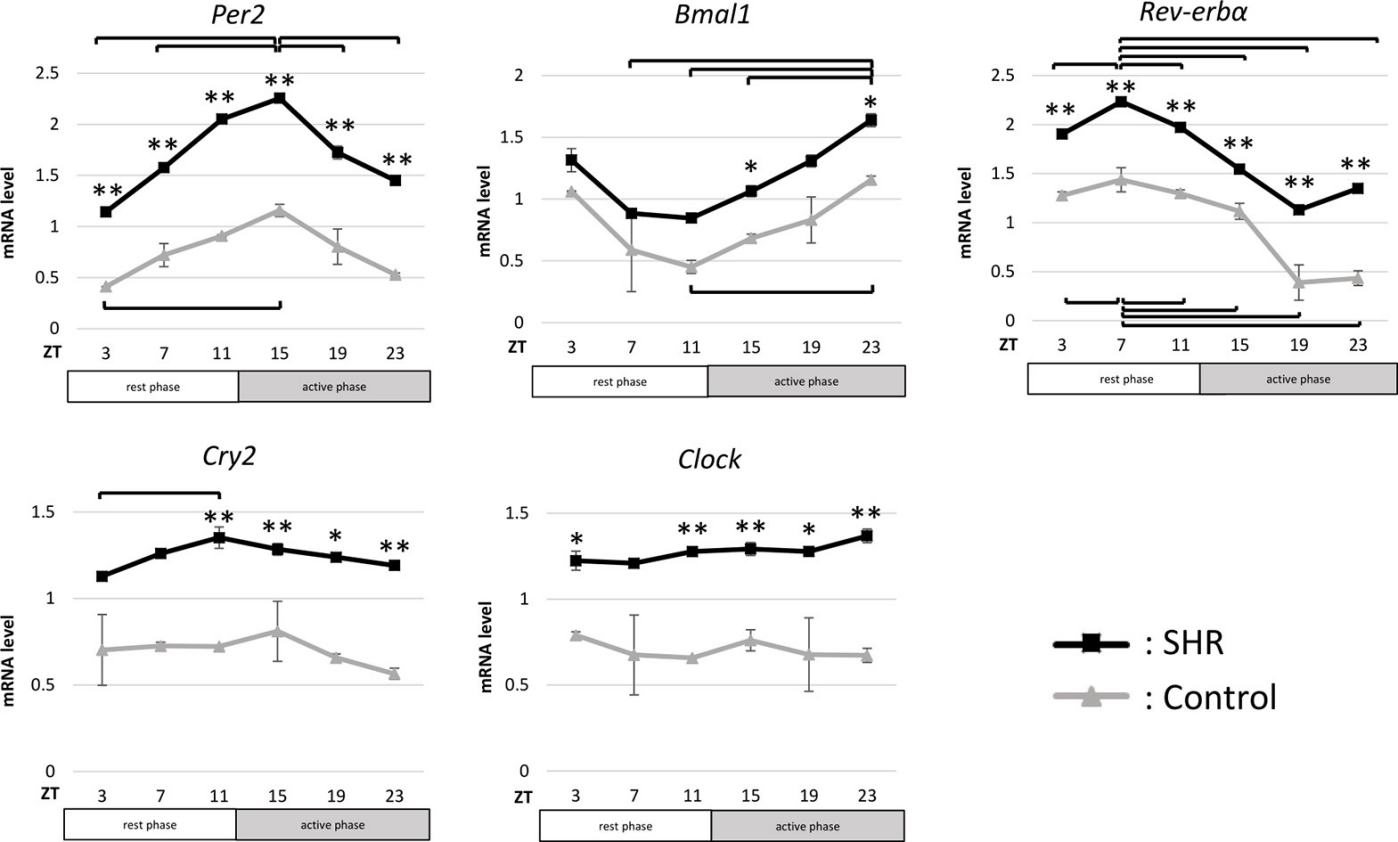
Changes in clock gene mRNA expression in bladders from Wistar rats (control) and spontaneously hypertensive rats (SHR) bladder. *Per2*, *Bmal1*, *Rev-erbα*, *Cry2*, and *Clock* mRNA expression rhythms in the bladder in control and SHR under 12-h light/12-h dark cycle. n = 6 for control, n = 6 for SHR at each time point. Statistic analyses were performed using a one-way analysis of variance. *p<0.05, ** p<0.01. ZT: zeitgeber time; *Per2*: period 2; *Bmal1*: brain and muscle aryl hydrocarbon receptor nuclear translocator-like protein 1; *Rev-erbα*: nuclear receptor subfamily 1, group D, member 1; *Cry2*: cryptochrome 2; *Clock*: circadian locomotor output cycles kaput.

In the SHR bladder, all five clock genes (*Per2*, *Bmal1*, *Rev-erbα*, *Cry2*, and *Clock*) showed high mRNA levels, especially *Per2* and *Rev-erbα*, which were both significantly higher at all time points compared to the control group (Fig 2). Circadian rhythms associated with *Per2*, *Bmal1*, and *Rev-erbα* expression were observed and showed similar variations between control and SHR groups. In both groups, *Per2*, *Bmal1*, and *Rev-erbα* expression had circadian rhythms with peaks and nadirs at ZT 15 and 3 for *Per2*, 23 and 11 for *Bmal1*, and 7 and 19 for *Rev-erbα*, respectively. On the other hand, *Cry2* and *Clock* mRNA levels of the SHR bladder were significantly higher in the active phase compared to the control group, although *Cry2* mRNA level at ZTs 3 and 7 (rest phase) and *Clock* mRNA level at ZT 7 (rest phase) were not significantly higher compared to the control group.

### Mechanosensors and VNUT gene expression

**Fig 3:**
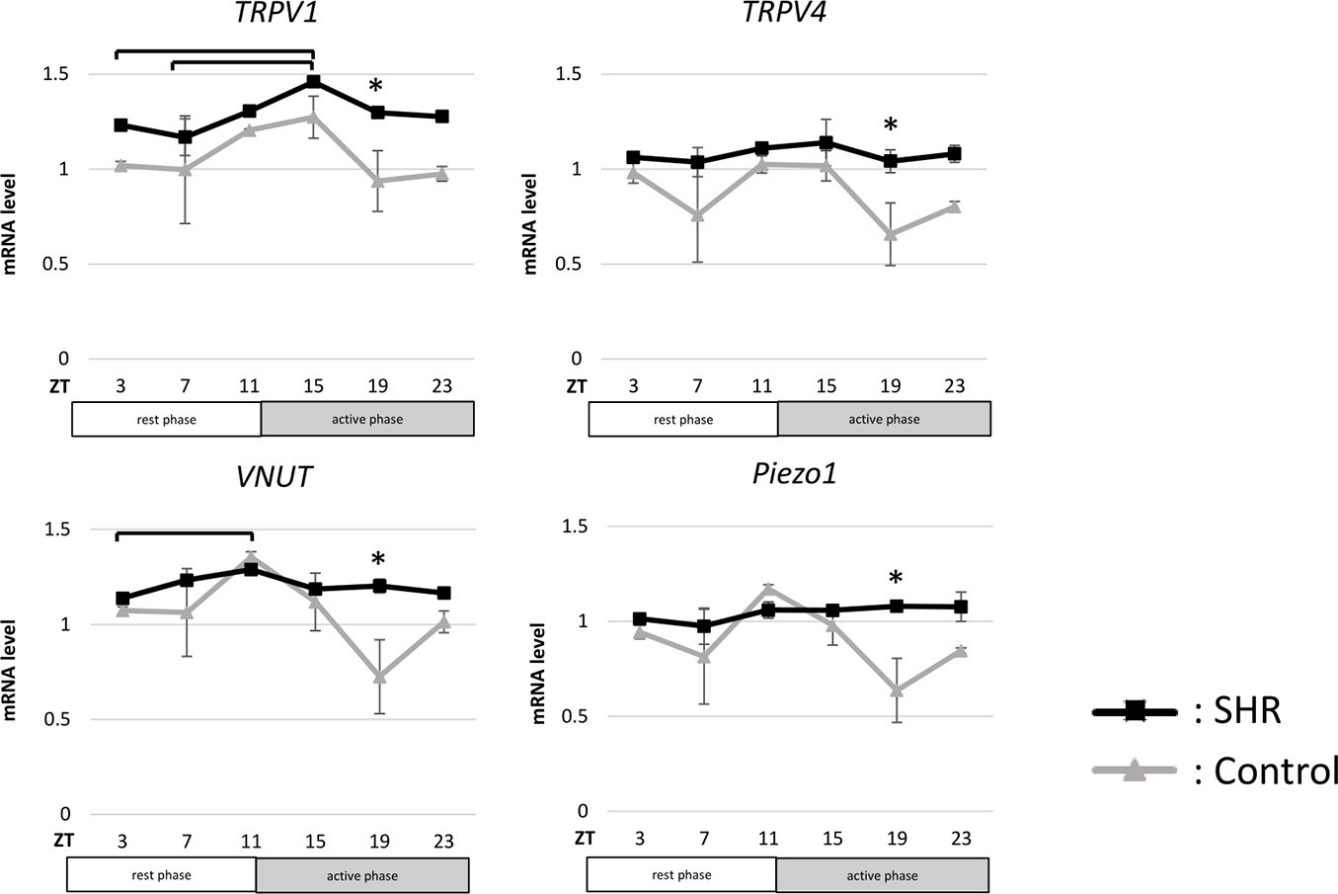
Changes in TRPV1, TRPV4, VNUT and Piezo1 mRNA expression in bladders from Wistar rats (control) and spontaneously hypertensive rats (SHR) bladder. *TRPV1*, *TRPV4*, *VNUT*, and *Piezo1* mRNA expression rhythms in the bladder in control and SHR under 12-h light/12-h dark cycle. n = 6 for control, n = 6 for SHR at each time point. Statistic analyses were performed using a one-way analysis of variance. *p<0.05, ** p<0.01. ZT: zeitgeber time; *TRPV1*: transient receptor potential vanilloid channel 1; *TRPV4*: transient receptor potential vanilloid channel 4; *VNUT*: vesicular nucleotide transporter.

In the SHR group, *TRPV1*, *TRPV4*, *Piezo1*, and *VNUT* mRNA levels were significantly higher at ZT 19 compared to the control group (Fig 3). In the control group, *TRPV1* mRNA level peaked at ZT 15, and that of *TRPV4*, *Piezo1*, and *VNUT* peaked at ZT 11, and ZT 11 peaks were observed to correspond to the rest/active phase change points. Furthermore, mRNA levels of *TRPV1*, *TRPV4*, *Piezo1*, and *VNUT* in control tended to decline at ZTs 7 and 19, between rest and active phase change points, and they showed a W-shaped expression pattern. On the other hand, mRNA levels of *TRPV1*, *TRPV4*, *Piezo1*, and *VNUT* in SHR fluctuated less than the control and showed a more flattened expression pattern.

## Discussion

To our knowledge, the present study is the first to examine the relationship between clock gene expression and voiding dysfunction in the SHR model. The results revealed that SHR group rats had a higher 24-h voiding frequency throughout the day but lower urine volume per void that did not fluctuate with daily phase. In addition, SHR also had higher mRNA levels of two bladder clock genes (*Cry2* and *Clock*).

The results of cystometric studies conducted by Inoue *et al*. in accordance with those of the present study revealed that urine volume per voiding, intercontractile intervals, and bladder compliance were significantly smaller in SHR than Wistar rats [21]. Stratification of urination measurements by phase in the current study revealed significantly higher voiding frequency and lower urine volume per voiding for SHR in both rest and active phases. In contrast, the control rats showed a significant difference in urine volume per voiding between rest and active phases, in agreement with the results of a report by Herrera *et al*. in Sprague Dawley rats [19]. According to their report, rat activity decreased during the light period as rats slept, only waking up to urinate, while it increased during the dark period along with decreased bladder capacity and increased urination frequency. These results clearly illustrate a light/dark (i.e., day-night) difference in bladder capacity and micturition frequency in chronically-instrumented nocturnal rodents that are phase-locked to the normal circadian locomotor activity rhythm of the animal. On the other hand, the results of the present study did not show a significant difference in urine volume per void (i.e., bladder capacity) in SHR between rest (light) and active (dark) phases.

Of the many types of clock genes identified to date, *Clock*, *Bmal*, *Cry*, and *Per* have been shown to play the most important roles in the regulation of circadian rhythm. Circadian *Bmal1* expression appears to be positively controlled by Per and Cry proteins, and transfection studies suggest that Bmal1 transcription may be negatively autoregulated by Bmal1 and Clock [22]. These results can be explained by the effects of overexpression of these proteins on Rev-erbα expression. Cycle accumulation of Rev-erbα then imposes circadian regulation on Bmal1 transcription. Some studies have reported the circadian expression of clock genes in the urinary bladder. Negoro *et al*. reported the circadian expression of *Clock*, *Per1*, *Per2*, *Cry1*, and *Bmal1* in the urinary bladder of wild-type mice, while dysfunction of the bladder circadian clock in *Cry*-null mice was associated with disturbed *Per2* and *Bmal1* expression rhythms [6]. Ihara *et al*. reported disrupted circadian expression of *Per2*, *Cry1*, *Bmal1*, and *Rev-erbα* mRNA in the bladder mucosa of *Clock*-mutant versus wild-type mice [23]. However, it is unclear whether phase of clock gene expression in mice bladders are similar to those seen in the rats. In this study, *Cry2* and *Clock* mRNA levels were significantly higher in SHRs in the active phase compared to control. Thus, the abnormal Cry2 and Clock circadian expression reported here may be one of the factors contributing to the bladder dysfunction observed in the SHR. However, further research will be necessary to test this prediction and to elucidate the precise mechanisms.

In the present study, mRNA levels of *TRPV1*, *TRPV4*, *Piezo1*, and *VNUT* in the SHR group during the active phase were found to be significantly higher compared to those in the control group. Ihara *et al*. reported disturbed diurnal variation of *TRPV4*, *VNUT*, and *Piezo1* expression in the bladder mucosa of *Clock*-mutant versus wild-type mice [23], and all three are known to be regulated by clock genes. It is tempting to speculate that increased expression of Cry2 and Clock in SHR during the active phase may be linked with expression levels of TRPV1, TRPV4, Piezo1, and VNUT, thereby lowering the threshold of stretch stimulus sensing. In turn, this is presumed to cause an increase in voiding frequency and a decrease in urine volume per void. Furthermore, a shallower, rhythmic expression pattern of TRPV1, TRPV4, *Piezo1*, and *VNUT* in SHR would be likely to lead to the disappearance of the daily fluctuation of urine volume per void.

In the present study, there are some limitations. First, urodynamic studies were not performed. Second, protein expression was not investigated. The path from mRNA translation into protein is complicated, and it has been previously reported that there is a 6-h interval between mRNA expression of peripheral tissues to clock protein expression in mouse liver [24]. Although it has been reported that mRNA and protein rhythms in the central nervous system [25] and urinary bladder mucosa [7] are almost simultaneous. The time difference between mRNA and protein expression under SHR pathology is unknown. Thus, in future studies a clear priority will be to determine the clock protein expression patterns in SHR versus control bladder tissue.

## Conclusions

In conclusion, from our present findings we speculate that increased expression of Cry2 and Clock in SHR during the active phase may be one of many possible factors contributing to the increase in urination frequency and the decrease in urine volume per voiding by increasing expression of *TRPV1*, *TRPV4*, *Piezo1*, and *VNUT*. However, additional functional experiments will be needed to test whether this interpretation is correct.

## Supporting information

S1 Table Results of body and bladder weight. Body and bladder weight in 18-week-old Wistar rats (control) and spontaneously hypertensive rats (SHR). Data are mean ± SEM. * Significantly different from the control group (p<0.01).
(XLSX)

S2 Table Results of voiding behaviors. Voiding frequency, voided volume and uine volume per void in the rest and active phase in 18-week-old Wistar rats (control) and spontaneously hypertensive rats (SHR) under 12-h light/12-h dark cycle. Data are mean ± SEM. * Significantly different from the control group (p<0.01). † Significantly different from rest phase (p<0.05). ‡ Significantly different from rest phase (p<0.01).
(XLSX)

S3 Table Results of clock gene mRNA expression in bladders from Wistar rats (control) and spontaneously hypertensive rats (SHR) bladder. *Per2*, *Bmal1*, *Rev-erbα*, *Cry2*, and *Clock* mRNA expression rhythms in the bladder in control and SHR under 12-h light/12-h dark cycle. Data are mean ± SEM. * Significantly different from the control group (p<0.01). † Significantly different from the control group (p<0.05). ‡ Significantly different from the peak (p<0.05). ZT: zeitgeber time; *Per2*: period 2; *Bmal1*: brain and muscle aryl hydrocarbon receptor nuclear translocator-like protein 1; *Rev-erbα*: nuclear receptor subfamily 1, group D, member 1; *Cry2*: cryptochrome 2; *Clock*: circadian locomotor output cycles kaput.
(XLSX)

S4 Table Results of TRPV1, TRPV4, VNUT and Piezo1 mRNA expression in bladders from Wistar rats (control) and spontaneously hypertensive rats (SHR) bladder. *TRPV1*, *TRPV4*, *VNUT* and *Piezo1* mRNA expression rhythms in the bladder in control and SHR under 12-h light/12-h dark cycle. Data are mean ± SEM. * Significantly different from the control group (p<0.05). † Significantly different from the peak (p<0.05). ZT: zeitgeber time; *TRPV1*: transient receptor potential vanilloid channel 1; *TRPV4*: transient receptor potential vanilloid channel 4; *VNUT*: vesicular nucleotide transporter.
(XLSX)
