# Correction: The circadian rhythm of bladder clock genes in the spontaneously hypersensitive rat

**DOI:** 10.1371/journal.pone.0227321

**Published:** 2019-12-27

**Authors:** Yusuke Kimura, Masashi Honda, Ryo Sasaki, Tetsuya Yumioka, Hideto Iwamoto, Panagiota Tsounapi, Shuichi Morizane, Katsuya Hikita, Mitsuhiko Osaki, Futoshi Okada, Atsushi Takenaka

The word “hypertensive” is misspelled in the article title. The correct title is: The circadian rhythm of bladder clock genes in the spontaneously hypertensive rat. The correct citation is: Kimura Y, Honda M, Sasaki R, Yumioka T, Iwamoto H, Tsounapi P, et al. (2019) The circadian rhythm of bladder clock genes in the spontaneously hypertensive rat. PLoS ONE 14(7): e0220381. https://doi.org/10.1371/journal.pone.0220381
